# Yellow Fever

**Published:** 1901-03-23

**Authors:** 


### YELLOW FEVER.

In the medical section of the Pan-American Medical Congress held last month at Havana, a paper was read by Major Reed of the United States Army, detailing the results of an investigation undertaken by a commission of the Army Medical Staff, in regard to the origin and mode of transmission of yellow fever. The investigations were undertaken at an experimental sanitary station near the town of Quemados, Cuba. At this station, in strict quarantine, and with every source of infection absolutely excluded, they succeeded in infecting 85·71 per cent. of those individuals who were bitten by purposely contaminated mosquitos. The experiments suggest that mosquitos do not merely carry the specific infective agent in their proboscis, but that the parasite of this disease, as is the case with malaria, must undergo a definite cycle of development in the body of the mosquito before the latter becomes infective—a period which would seem to occupy not less than twelve days. They show also that house infection is due to the presence in the house of infected mosquitos, and that bedding contaminated by contact with yellow fever cases, or by the excreta of these patients, is absolutely without effect in conveying this disease. Yellow fever can be experimentally produced by the subcutaneous injection of blood taken from the general circulation during the first and second days of the disease. But an attack of yellow fever produced by the bite of an infected mosquito confers immunity against a subsequent injection of the blood of an individual suffering from the non-experimental form of disease. Lastly, although the mode in which yellow fever is propagated has now, it is held, been definitely determined, the specific cause of the disease, the nature of the parasite, whatever it may be, that is transmitted, has yet to be discovered.

